# Tanshinone IIA induces cell death via Beclin‐1‐dependent autophagy in oral squamous cell carcinoma SCC‐9 cell line

**DOI:** 10.1002/cam4.1281

**Published:** 2018-01-06

**Authors:** Ye Qiu, Conghua Li, Qinhua Wang, Xingqi Zeng, Ping Ji

**Affiliations:** ^1^ Stomatological Hospital of Chongqing Medical University North Songshi Road #426 Chongqing 401147 China; ^2^ Chongqing Key Laboratory of Oral Diseases and Biomedical Sciences North Songshi Road #426 Chongqing 401147 China; ^3^ Chongqing Municipal Key Laboratory of Oral Biomedical Engineering of Higher Education North Songshi Road #426 Chongqing 401147 China

**Keywords:** Akt, Atg12‐Atg5, Atg7, autophagy, Beclin‐1, mTOR, oral squamous cell carcinoma, PI3K, tanshinone IIA

## Abstract

Tanshinone IIA (TAN) is one of the major functional compounds of *Salvia miltiorrhiza* Bunge and possesses the ability to suppress the growth of multiple cancer cell types via its apoptosis‐ and autophagy‐inducing functions. In this study, the effect of TAN therapy on the survival of oral squamous cell carcinoma (OSCC) was evaluated, and the underlying mechanism involved in the treatment was investigated. Human oral squamous cell carcinoma cell SCC‐9 was used for in vitro assays and induction in an OSCC xenograft mouse model. The tumor cells were subjected to TAN administration at different concentrations. Then the apoptosis and autophagy processes in SCC‐9 cells were evaluated and the activities of Beclin‐1/Atg7/Atg12‐Atg5 and PI3K/Akt/mTOR pathways were determined. In addition, by knocking down the expression of *Beclin‐1* in SCC‐9 cells, the study also assessed the role of the indicator in the anti‐OSCC effect of TAN. Results of in vitro assays were further validated with an OSCC xenograft mouse model. Administration of TAN‐induced cell apoptosis and upregulated the expression of cleaved‐caspase‐3. Simultaneously, the autophagy process in SCC‐9 cells was initiated by TAN, which was signaled by the formation of autophagosomes and increase in the ratio of LC3 II/LC3I. The above processes were associated with the activation of Beclin‐1/Atg7/Atg12‐Atg5 signaling and inhibition of PI3K/Akt/mTOR signaling. Our results also inferred a partially Beclin‐1‐dependent mechanism of action of TAN in OSCC cells: knockdown of the *Beclin‐1* blocked the effect of TAN on SCC‐9 cells both in vivo and in vitro. Our study provided a preliminary explanation of the mechanism involved in TAN effect: the agent exerted its autophagy‐inducing effect against OSCC in a multipronged manner, by both inducing the Beclin‐1/Atg7/Atg12‐Atg5 pathway and suppressing the PI3K/Akt/mTOR pathway.

## Introduction

Oral squamous cell carcinoma (OSCC) is the most frequently diagnosed primary tumor in the oral cavity, accounting for *ca*. 90% of all the oral cancer cases 1. Human OSCC is largely associated with the risk factors including tobacco, areca nut and alcohol exposure. Growing evidence has also shown that chronic dental trauma, poor oral hygiene, and infection with human papillomavirus are contributing factors for oncogenesis of OSCC 2, 3, 4. In addition, the susceptibility of an individual to oral cancer is closely associated with specific genetic factors 5, 6. Although the current treatments with surgery, external beam radiation, and brachytherapy for oral cancer are widely used, these treatment modalities are sometimes inadequate, and local invasion and metastases may develop in a considerable proportion of OSCC patients 7. Therefore, to promote the treatment efficacy of OSCC, numerous studies have been performed to explore novel targets for the development of promising new anticancer drugs.

Among the studies aiming to identify novel therapeutic targets, those focusing on the autophagy process of cancer cells have achieved considerable progress in recent years 8, 9, 10. Characterized by sequestration of cellular organelles and proteins into autophagic vesicles 8, basal autophagy is critical for the housekeeping of the cell, which disposes waste via removal of damaged cellular organelles and protein aggregates. Under stress conditions, autophagy supplies the cell with an internal source of nutrients and helps cells adapt and survive in the face of unfavorable conditions. Given the well‐known role of autophagy in normal physiology, emerging evidence suggests that autophagy in different types of cancers plays a dual role, either suppressing early carcinogenesis or supporting survival of advanced tumors 11. Regarding OSCC, Kim and colleagues reported that curcumin‐induced autophagy decreased the survival of oral cancer cells 12. Their work inspired the development of autophagy‐based anti‐OSCC therapies, and many natural products have been shown to potentially induce autophagy in vitro 12, 13.

Among the numerous natural products studied for their anticancer potential, the dried root or rhizomes of *Salvia miltiorrhiza* Bunge (namely, Danshen or Tanshen) has long been used in Traditional Chinese Medicine (TCM) and Eastern countries in preventive or therapeutic remedies for coronary heart diseases and vascular diseases 14, 15. Since 1930, more than 90 chemical constituents have been identified from Danshen 16, and a large proportion of these compounds exhibit the potential for broad anticancer properties in cell culture models 15, 17, 18. In this respect, Tanshinone IIA (TAN) is one of the most extensively studied. As the major functional compound of Danshen, TAN has been shown to antagonize the proliferation of multiple human cancer cell lines, such as human hepatocellular carcinoma cells, human non–small cell lung cancer, and human promyelocytic leukemia cell 19, 20. Moreover, Ding et al. also reported that incubation with TAN could sensitize OSCC to radiation by inducing autophagy. Given the role of autophagy itself in the development of anticancer therapies, it was deemed appropriate to comprehensively explore the effect of sole TAN administration on the autophagy process in OSCC cells as well as the mechanism driving the treatment.

Therefore, in this study, a human OSCC SCC‐9 and a SCC‐9 xenograft mouse model were employed as in vitro and in vivo research systems. The effect of TAN administration on tumor growth both in vitro and in vivo was firstly assessed. The mechanism involved autophagy‐dependent cell death. Additionally, the central modulator of autophagy, *Beclin‐1*, was knocked down in SCC‐9 cells to further verify the regulation pattern of TAN on autophagy. Findings outlined in this study showed that TAN administration was lethal to SCC‐9 cells, which took action by inducing autophagy via a Beclin‐1‐dependent manner.

## Materials and Methods

### Animals and cell cultures

Human oral squamous cell carcinoma SCC‐9 cells were purchased from Cancer research Institute of Southern Medical University and cultured in Minimum Essential Medium (MEM) (Invitrogen, Carlsbad, CA) supplemented with 10% fetal bovine serum (FBS) (Hyclone, Logan, UT) and 2 mmol/L glutamine at 37°C in a humidified atmosphere of 5% CO_2_. Cells passaged three to five times were used for subsequent assays. BALB/c‐nu mice purchased from Experimental Animal Center (Chongqing medical university) were used for in vivo tumor induction experiments and maintained in cages at room temperature (20–25°C) with constant humidity (55 ± 5%) and provided food and water ad libitum. All animal assays were performed following the Institutional Animal Ethics Committee and Animal Care Guidelines for the Care and Use of Laboratory Animals of Chongqing medical university.

### Chemicals

TAN with high purity (over 98%) was purchased from MeilunBio (Dalian, China). Antibodies against cleaved‐caspase‐3, LC3‐I/II, Beclin‐1, Atg‐7, Atg12/Atg5, PI3K, phosphorylated PI3K (p‐PI3K), PDK1, p‐PDK1, Akt, mTORC1, and p‐MTOR C1 were purchased from Abcam.

### Determination of IC50 of TAN on SCC09 cells

The IC50 of TAN was determined using the Cell Counting Kit‐8 (CCK‐8) assay: briefly, 50 *μ*L exponentially growing SCC‐9 cells at concentration of 2 × 10^5^ cells/mL were seeded into each well of a 96‐well plate and incubated with different concentrations of TAN (0, 2, 4, 8, 16, 32, 64 *μ*mol/L; each concentration was represented by three replicates.). After incubation, CCK‐8 solution (10 *μ*L) was added to each well and the cultures were incubated at 37°C for 90 min. The OD values in different wells were determined with a Mircoplate Reader at 450 nm. IC50 was calculated using a logit model, and IC50 of TAN in inhibiting SCC9 cells was 17.54 *μ*mol/L.

### Knockdown of Beclin‐1 in SCC09 cells

Beclin‐1 specific (5′‐ ACUUUCUGUGGACAUCAUCCU ‐3′) and non‐targeting (5′‐ ACGUGACACGUUCGGAGAATT‐3′) siRNAs were synthesized by Sangon Biotech (Shanghai, China). Sequences of different siRNAs were ligated into pU6 plasmid to form transfection vectors. Transfection was conducted using transfection agents of Applygen Technologies Inc. (No. c1507, Beijing, China) according to the manufacturer's instruction. Stable transfected cells for further experiments were screened in medium with the presence of G418 (0.5 *μ*g/*μ*L).

### Tumorigenicity assay

Healthy BALB/c‐nu mice were subcutaneously injected in the back with 0.2 mL (5 × 10^7^/mL) SCC‐9 cells or sh‐Beclin‐1 plasmid‐transfected SCC09 cells to induce the formation of solid tumor in vivo.

### Experimental designs

To comprehensively investigate effect of TAN administration on autophagy in SCC‐9 cells and deduce the causative mechanism, three independent experiments were designed. First, four groups were set up to evaluate the effect of TAN on SCC‐9 survival and autophagy‐related signaling: (A) a Blank group of SCC‐9 cells (B) a 0.5 IC50 group, consisting of SCC‐9 cells incubated with 0.5‐fold IC50 concentration TAN for 24 h, (C) an IC50 group, consisting of SCC‐9 cells were incubated with IC50 concentration TAN for 24 h, and (D) a 2 IC50 group, consisting of SCC‐9 cells incubated with twofold IC50 concentration for 24 h. To validate the key role of Beclin‐1 in autophagy induced by TAN, five groups were set up: (A) a Blank group of SCC‐9 cells, (B) a TAN group, consisting of SCC‐9 cells incubated with IC50 concentration TAN for 24 h, (C) an siRNA group, consisting of *Beclin‐1* knockdown SCC‐9 cells, (D) an siRNA+TAN group, consisting of *Beclin‐1* knockdown SCC‐9 cells incubated with IC50 concentration TAN for 24 h, and (E) a CQ group, consisting of SCC‐9 cells incubated with chloroquine for 24 h. For in vivo assays, 18 BALB/c‐nu mice were randomly divided into three groups: (A) a Blank group of OSCC mice, (B) a TAN group, consisting of OSCC mice subcutaneously injected with TAN, and (C) a siRNA+TAN group, consisting of Beclin‐1 knockdown OSCC mice subcutaneously injected with TAN. The mice were raised under the same conditions for 21 days. The volume, major axis, and minor axis of the solid tumors were measured every 3 days starting the day tumor could be observed with the naked eye. Upon completion of the assay, all of the mice were sacrificed using the air embolism method and tumor tissues were harvested and preserved at –80°C for subsequent assays.

### Flow cytometry

Cells in different groups were collected with centrifugation at 300 ×g for 5 min, and apoptotic rates were determined using an Apoptosis Detection Kit (Catl. No. KGA106, KeyGEN BioTECH, Nanjing, China) according to the instructions for manufacturers. Briefly, 5 *μ*L Annexin V was added to each well. After incubation with Annexin V for 10 min at room temperature, the cells were resuspended with 1 ×  Binding buffer, and 5 *μ*L propidium iodide (PI) was added. After a 15‐min incubation in the dark, the apoptotic rates were analyzed using a FACScan flow cytometer (Accuri C6, BD). The apoptotic cell rate (UR + LR–all apoptosis cell percentage) was equal to the sum of the late apoptotic rate (UR, upper right quadrant–advanced stage apoptosis cell percentage) and the early apoptotic rate (LR, lower right quadrant–prophase apoptosis cell percentage).

### Transmission electronic microscope

SCC‐9 cells were harvested and fixed with 2.5% glutaraldehyde in PBS (pH 7.8) for 2 h at room temperature and then in 0.1% osmium tetroxide for 1 h at 4°C. After dehydration with a series of graded ethyl alcohols, cells were embedded in epoxy resin and sectioned. The 50–60 nm ultrathin sections were loaded on copper grids and stained with uranyl acetate and lead citrate. Autophagosome morphology was observed with transmission electronic microscope (TEM) (JEM‐1230, Japan Electron Optics Laboratory, Japan) at 20,000× magnification.

### Immunofluorescent assay

For immunofluorescent detection, autophagosomes isolated from different groups were fixed with 4% paraformaldehyde for 15 min and permeabilized with 0.1% Triton X‐100 for 30 min. After being washed with PBS for three cycles (5 min per cycle), the slides were blocked in 10% goat serum for 15 min. Primary rabbit polyclonal antibody against LC3B (ab48394, Abcam, English) was then added and the slides were incubated overnight at 4°C. Staining was performed by incubating the cells with secondary antibody (ab150075, Abcam) for 1 h in dark. After incubation with the secondary antibody, slides were washed and stained with 4,6‐diamino‐2‐phenyl indole (DAPI) for 5 min at room temperature and then visualized with a fluorescent microscopy (BX53, OLYMPUS) at 400× magnification.

### Western blotting assay

Total protein product in different cell and solid tumor samples was extracted using the Total Protein Extraction Kit according to the manufacturer's instructions (Catalog No. WLA019, Wanleibio, China). Glyceraldehyde 3‐phosphate dehydrogenase (GAPDH) was used as internal reference protein. Concentrations of protein samples were determined using the bicinchoninic acid (BCA) method, and 40 *μ*g of protein was subjected to a 10% sodium dodecylsulfate polyacrylamide gel electrophoresis (SDS‐PAGE). After transferring the target proteins onto polyvinylidene difluoride (PVDF) sheets, the membranes were washed with TTBS for 5 min and incubated with 5% (M/V) skim milk powder solution for 1 h. Primary antibodies against target proteins, including LC3 I/II (ab48394), Beclin‐1 (ab210498), Atg7 (ab108338), Atg12/Atg5 (AAM79, AbD Serotec, USA), PI3K (ab40755), p‐PI3K (ab182651), Akt (ab18785), pAkt (ab81283), mTOR C1 (ab2732), p‐MOR C1 (ab109268), PDK1 (ab186870), p‐PDK1 (ab32800), or GAPDH (1:1000) were incubated with membranes 4°C overnight. After additional four washes using TTBS, secondary HRP IgG antibody (ab7090) was added and incubated with the membranes for 45 min at 37°C. After another six washes using TTBS, the blots were developed using Beyo ECL Plus reagent and the results were recorded in the Gel Imaging System. The relative expression levels of proteins in different samples were calculated with Gel‐Pro‐Analyzer (Media Cybernetics, USA).

### H&E staining

The histological changes in tumor tissues of different groups were observed using H&E staining: tissues were placed into Bouin solution (4% formaldehyde) for perfusion fixation. They were then dehydrated using different concentrations of alcohol and vitrified in dimethylbenzene. Samples were embedded in paraffin, sectioned and stained with H&E and the results were visualized under a microscope at 400× magnification. Following H&E staining, the nuclei in tissue were stained blue, and cytoplasms were stained red.

### Immunohistochemistry

For the immunohistochemical assay, tumor sections were incubated at 60°C for 2 h before incubation with dimethylbenzene for dewaxing. The sections were then hydrated with alcohol and washed with ddH_2_O for 2 min. Subsequently, the sections were fixed using 3% H_2_O_2_ for 15 min and washed with PBS for three times, followed by incubation with a primary antibody against Beclin‐1 (ab210498) and LC3 I/II (ab48394)at 37°C for 30 min and then at 4°C overnight. After three 5‐minute washes with 0.01 mol/L phosphate‐buffered saline (PBS), a secondary antibody (ab7090) was added to the sections for 30 min incubation at 37°C, followed by five washes with PBS. Thereafter, the sections were incubated with horse radish peroxidase (HRP)‐labeled avidin for 30 min at 37°C, and reacted with 3,3′‐diaminobenzidine (DAB) for 3–10 min before the reaction was stopped by ddH_2_O. The sections were restained with hematoxylin and dehydrated. Aperio ImageScope software (Aperio Technologies, Vista, CA) was used to evaluate the scanned images based on the percentage of positively stained cells and the staining intensity.

### Statistical analysis

All the data were expressed in the form of mean±SD. Normal distribution data and repeated measurement experiments were analyzed using ANOVA analysis. Significance was accepted with *P *<* *0.05. All statistical analyses and graph manipulations were conducted using SPSS version 19.0 (IBM, Armonk, NY).

## Results

### Determination of IC50 concentration for TAN treating SCC‐9 cells

**Figure 1:**
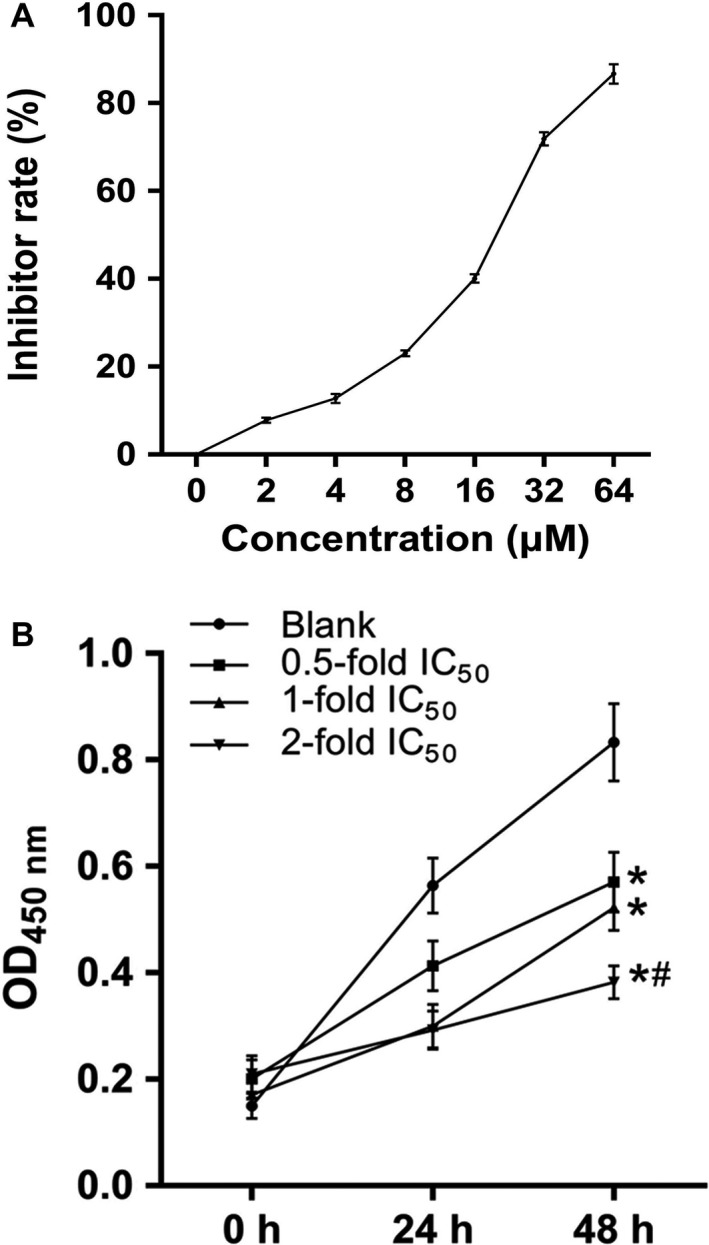
Administration of TAN suppressed the cell viability of SCC‐9 cells. (A) Determination of IC50 of TAN in treated SCC‐9 cells. (B) TAN decreased the cell viability of SCC‐9 cells as represented by OD450, and the effect was dose‐dependent. “*”, significantly different from Blank group, *P *<* *0.05. “#”, significantly different from 0.5‐fold group, *P *<* *0.05.

IC50 concentration for TAN to treat SCC‐9 cells was determined with CCK‐8 assay. As shown in Figure 1A, the inhibition rate of TAN on SCC‐9 cells increased with the incubating concentration. Using a logit model, the IC50 was determined as 17.54 *μ*mol/L, which was employed for subsequent in vitro assays. Moreover, the inhibiting effect of TAN on SCC‐9 cell growth was dose‐dependent: inhibition rate significantly decreased when SCC‐9 cells were incubated with twofold IC50 compared with the other two concentrations (Fig. 1B) (*P *<* *0.05).

### TAN promoted apoptosis in SCC‐9 cells

**Figure 2:**
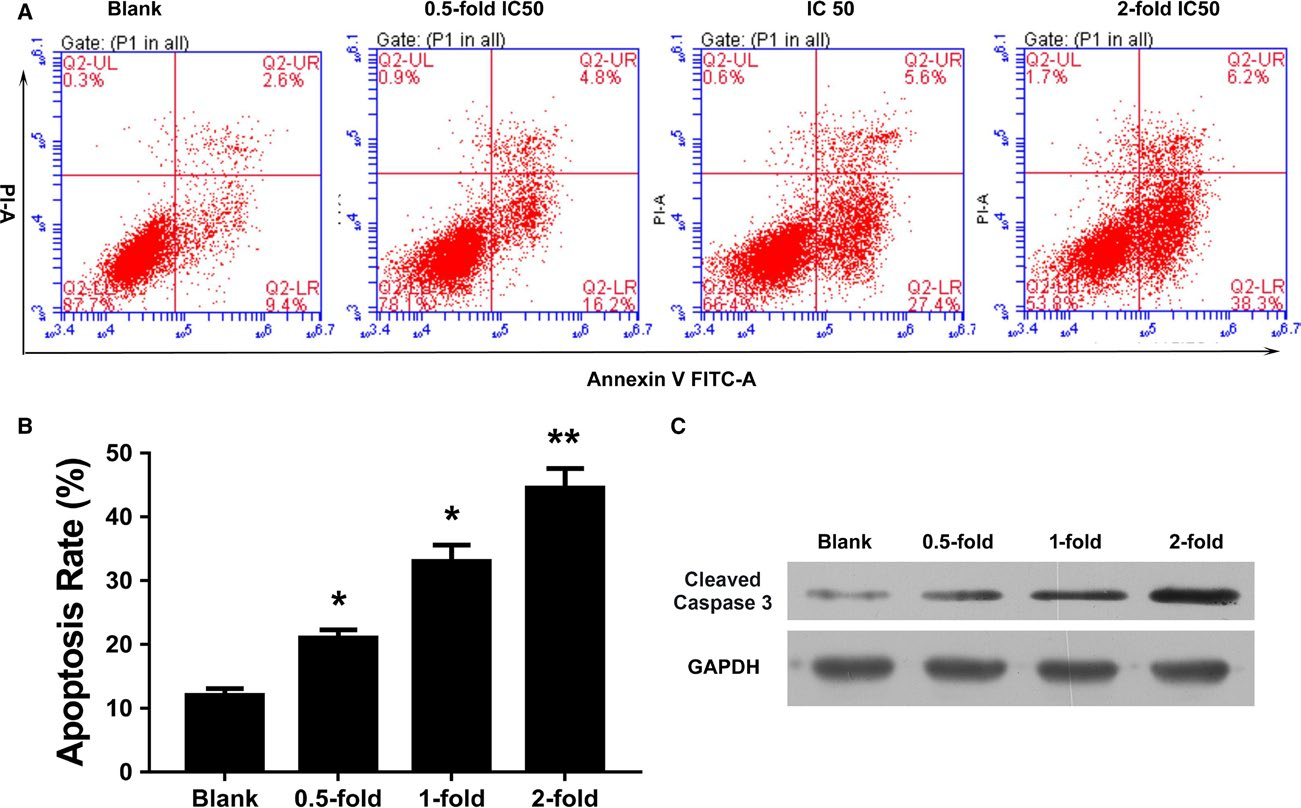
Administration of TAN‐induced apoptosis in SCC‐9 cells, and the effect was dose‐dependent. (A) and (B) Representative images of flow cytometry validation. (C) Representative images of Western blotting assay of cleaved‐caspase‐3.

After exposure to different concentration of TAN, SCC‐9 cells were subjected to flow cytometry to detect apoptosis. Results in Figures 2A and B showed that administration of TAN at all concentrations tested led to increased apoptosis rates compared with the Blank group. Moreover, with Western blotting detection, it was found that the activity of cleaved‐caspase‐3 was induced after administration of TAN (Fig. 2C). For both detections, the function of TAN was dose‐dependent, with twofold IC50 concentration of TAN exerting the strongest effect.

### Administration of TAN‐induced autophagy‐dependent cell death in SCC‐9 cells

**Figure 3:**
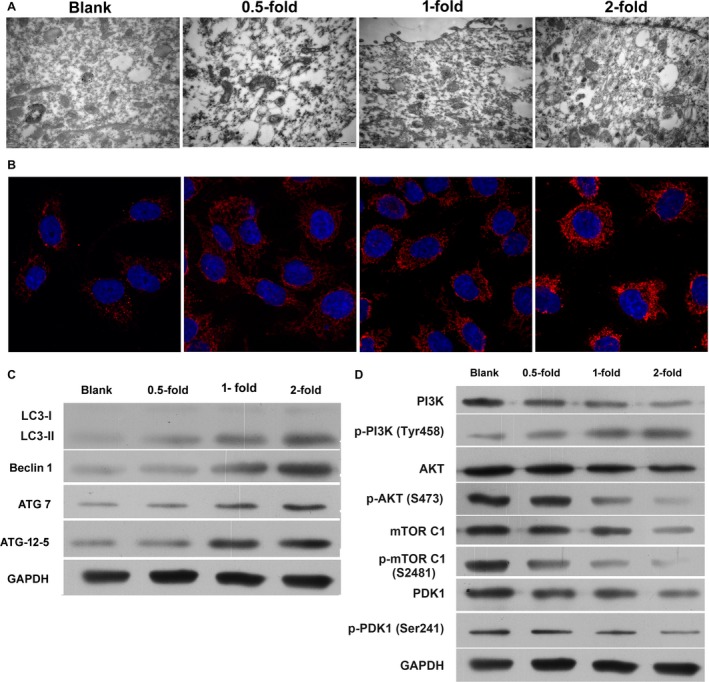
Administration of TAN‐induced autophagy‐dependent cell death in SCC‐9 cells and the effect was dose‐dependent. (A) Representative image of TEM of autophagosomes at 20,000× magnification, with readily visibly double‐membrane autophagic bodies engulfing many cellular organelles and macromolecules. Scale bar, 500 nm. (B) Representative images of immunofluorescent assay of LC3B (stained red). (C) Representative images of indicators associated with autophagy. Signaling of Beclin‐1/Atg7/Atg12‐Atg5 was activated while signaling of PI3K/Akt/mTOR was inhibited.

In order to determine whether TAN induces autophagy, the ultrastructure of SCC‐9 after incubation with TAN was examined using TEM. Immunofluorescence staining on LC3B and transmission electron microscope were used to detected the autophagy autophagosome in Figure 3A and B. Accordingly, after incubation with different concentrations of TAN, visible double‐membrane autophagic bodies engulfing many cellular organelles, including nondegradable organelles and macromolecules could be observed. Moreover, a monolayer of autophagosomes was typically noted in TAN‐treated groups as well (Fig. 3A). To further confirm the autophagy‐inducing effect of TAN, the expression of LC3B, a typical indicator of autophagy, was detected with immunofluorescent assay. As shown in Figure 3B, the expression and distribution of LC3B (stained red) on the cell surface was clearly amplified by administration of TAN, and the effect was dose‐dependent (Fig. 3B).

The level of LC3 II/LC3 I is a hallmark of the degree of autophagy. The ratio between the two indicators was investigated with Western blotting assay. Similar to the expression of LC3B on cell surface, values of LC3 II/LC3 I increased with the concentration of TAN, representing an elevated degree of autophagy (Fig. 3C).

Beclin‐1 signaling plays a vital role in coordinating the cytoprotective function of autophagy and in opposing the cellular death process of apoptosis 9, 21. PI3K/Akt/mTOR is a signaling transduction that is critical in regulating cell cycle, cell apoptosis and autophagy 10. The interaction between the two pathways in determining autophagy was also validated previously 21. Therefore, the expression of key members involved in the two pathways was also evaluated in this study. The levels of proautophagy molecules, including Belclin‐1, Atg7, Atg12‐Atg5, and p‐PI3K were all induced by administration of TAN (Fig. 3C). Correspondingly, the activities of antiautophagy indicators, including PI3K, p‐Akt, m‐TOR C1, p‐MOR C1, and PDK1 were all suppressed by TAN. The effect of TAN on the expression of those indicators was dose‐dependent, confirming the autophagy‐inducing effect of TAN on SCC‐9 cells (Fig. 3C).

### The Beclin‐1‐dependent pathway was central to the autophagy‐inducing effect of TAN

**Figure 4:**
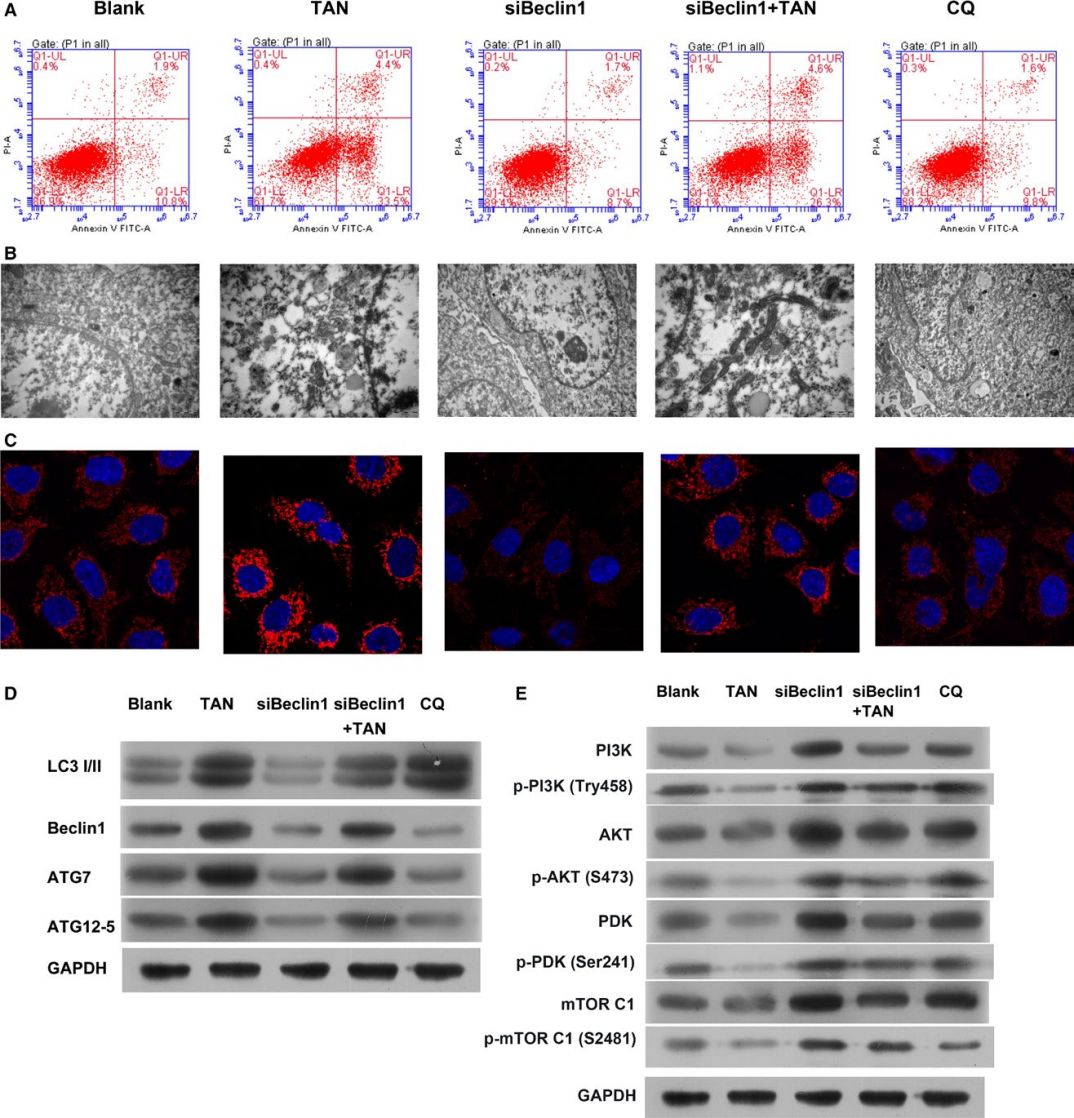
Knockdown of Beclin‐1counteracted the effect of TAN in antagonizing SCC‐9 cells. (A) Representative images of flow cytometry validation. (B) Representative images of TEM of autophagosomes. Scale bar, 500 nm. (C) Representative images of immunofluorescent assay of LC3B (stained red). (D) Representative images of indicators associated with autophagy. (E) The impact of TAN and Beclin1 on PI3K/AKT pathway were assessed by Western blot.

To further explore the mechanism driving the autophagy‐inducing function of TAN, expression of *Beclin‐1* in SCC‐9 cells was knocked down with specific siRNA. Compared with the Blank group, the cell death process in the siBeclin‐1 group was dramatically inhibited, and the effect was comparable to QC group. The apoptosis rates in the siBeclin‐1 and QC groups were reduced (Fig. 4A), and as shown in Figures 4B and C, the regular ultrastructure of SCC‐9 cells was maintained and the expression of LC3B was inhibited in the siBeclin‐1 and QC groups. The effect of *Beclin‐1* knockdown not only influenced the autophagy process in normal SCC‐9 cells but also counteracted the effect of TAN administration: the induced apoptosis process and autophagic deterioration of cell structure induced by TAN treatment was suppressed in *Beclin‐1* knockdown SCC‐9 cells (Fig. 4A–C). At the molecular level, the expression pattern of all the indicators in TAN group was reserved by *Beclin‐1* knockdown (Fig. 4D). Taken together, it was concluded that the autophagy‐inducing effect of TAN on SCC‐9 cells depended critically on the activity of Beclin‐1.

### Administration of TAN inhibited growth of solid tumor in vivo through a Beclin‐1‐dependent manner

**Figure 5:**
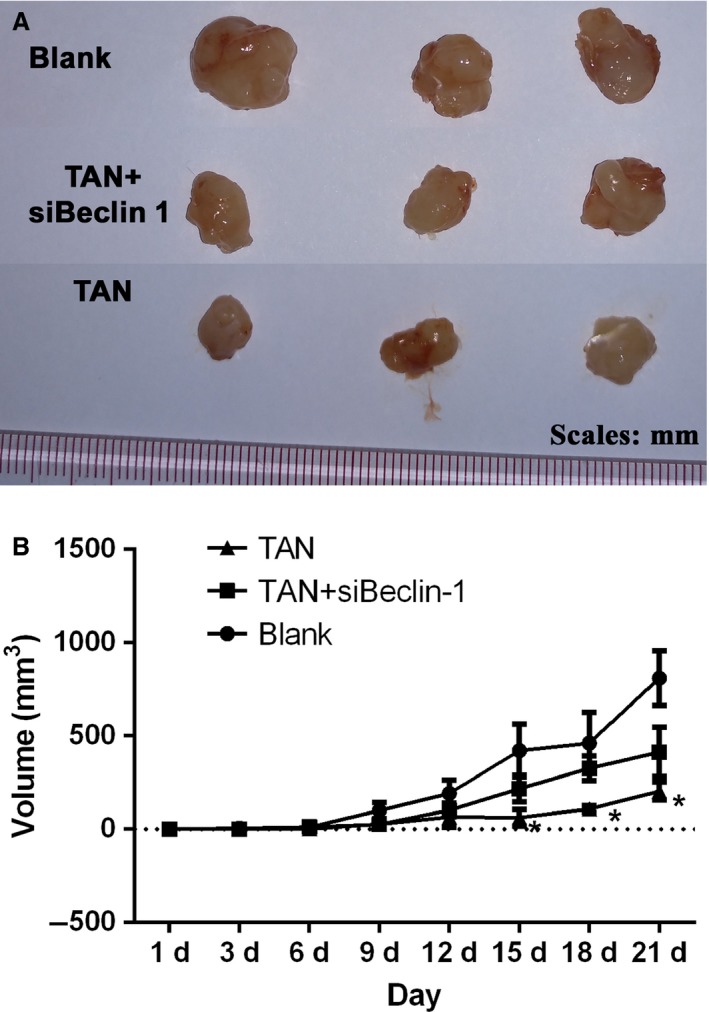
The growth‐inhibiting effect of TAN on solid tumors in vivo was weakened by knockdown of Beclin‐1. (A) The conditional SCC‐9 cells were subcutaneously injected into the rear flank of nude mice and treated with or without TAN. (B) The mean tumor size (mm^3^) was determined. “*”, significantly different from siRNA + TAN group, *P *<* *0.05.

**Figure 6:**
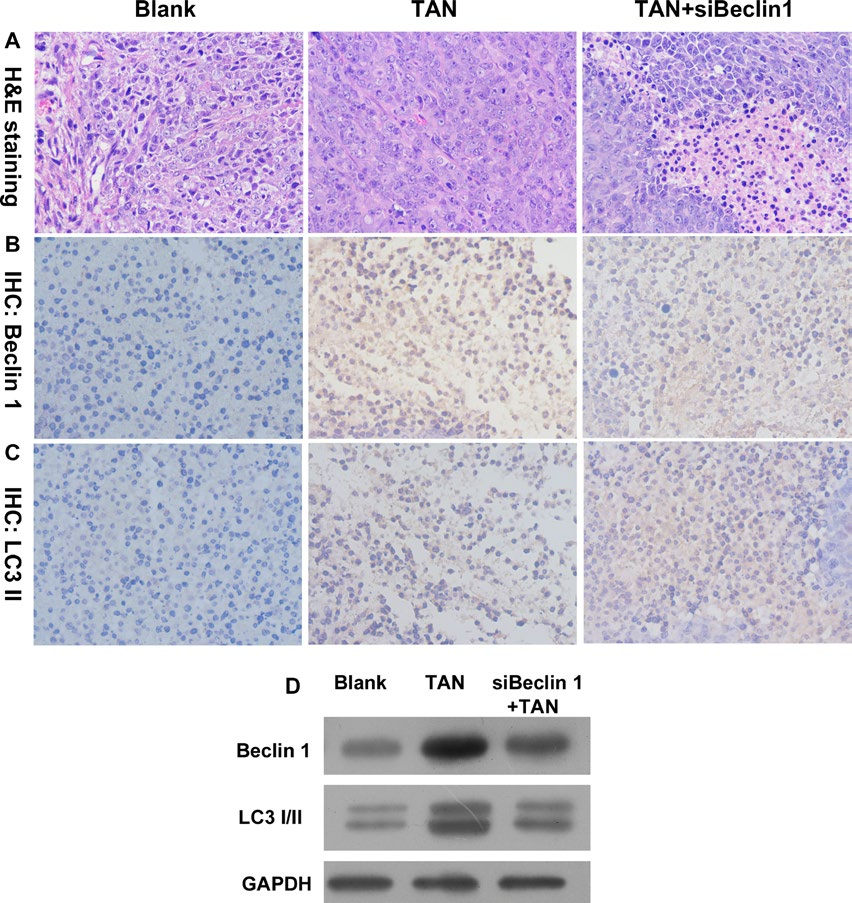
The autophagy process in OSCC was inhibited by knockdown of Beclin‐1. (A) Representative images of H&E staining. Magnification, 400×. (B) Representative images of immunochemistry of LC3B. Magnification, 400×. (C) Representative images of immunochemistry of Beclin‐1. Magnification, 400×. (D) Representative images of Western blotting of Beclin‐1 and LC3B.

The results of in vitro assays provided solid evidence of the autophagy‐inducing effect of TAN in SCC‐9 cells, and also suggested a Beclin‐1‐dependent mechanism of action. Based on the data, the treatment effect of TAN on OSCC was further investigated with a tumor xenograft animal model. As shown in Figure 5, administration of TAN suppressed the growth rate of solid tumors in SCID mice, and a significant difference between groups was observed starting on day 12 of the experiment (*P *<* *0.05). The in vivo assays also showed that administration of TAN significantly suppressed the growth of siBeclin‐1 knockdown tumor tissues when compared with Blank groups (Fig. 5) (*P *<* *0.05). In addition, H&E staining showed that administration of TAN‐induced cell structure deterioration in tumor tissues, and even with *Beclin‐1* knockdown, the effect was observed with H&E staining (Fig. 6A). The autophagy process in solid tumors was validated by immunochemistry of LC3B and Beclin‐1 as well as Western blotting of Beclin‐1, in which the expression of both indicators was upregulated by TAN administration (Fig. 6B–D), confirming the tumor‐suppressing effect of TAN in vivo.

## Discussion

As one of the most pharmacologically active phytochemicals isolated from Danshen, TAN is widely known to induce apoptosis and inhibit angiogenesis and metastasis in various cancer cells, including gastric, liver, leukemia, and lung cancers 22, 23, 24, 25, 26, 27, 28, 29. Recently, a study by Ding et al. reported the sensitizing effect of TAN on OSCC cells to radiotherapy by inducing the autophagy process in tumor cells 30. That study prompted us to investigate the effect of sole TAN administration on the survival of OSCC. Thus, in this study, a series of in vivo and in vitro assays was conducted to evaluate the potential of TAN alone as an alternative therapy against OSCC and to elucidate the underlying mechanism involved in this anti‐OSCC therapy.

By using the SCC‐9 cell line and an OSCC xenograft mice model, the OSCC‐suppressing effect of TAN was validated. Moreover, the inhibiting effect of TAN was dose‐dependent, as shown in CCK‐8 assays. In the in vitro tumorigenicity assay, the average solid tumor volume in the TAN group was only 25% of the volume in the Blank group. The two assays evidently verified the OSCC‐suppressing effect of TAN even without radiotherapy.

To explain the molecular mechanism associated with the anti‐OSCC function of TAN, the apoptosis and autophagy processes were investigated in this study. Flow cytometry showed an augmented apoptosis process in TAN‐treated SCC‐9 cells, which was associated with the upregulation of cleaved‐caspase‐3. The autophagy‐dependent cell death in SCC‐9 cells was also initiated by TAN administration. TEM observation illustrated the deterioration in cell structure, and immunofluorescent assay showed expanded expression and distribution of the autophagy indicator LC3B in autophagosomes. At the molecular level, LC3 II/LC3 I levels were increased by administration of TAN, which was a hallmark of initiation of autophagy within the tumor microenvironment 8. Although in some conditions, increased ratio of LC3 II/LC3 I was not associated with the increased with the autophagy‐related proteins Beclin‐1, Atg‐7, Atg12‐Atg5, all of those indicators were upregulated in this study, further confirming the autophagy‐inducing effect of TAN on SCC‐9 cells 8. Of all these indicators, Beclin‐1 is required for Atg7‐dependent autophagy 31. It has been well demonstrated that autophagy depends on the activity of Atg7 and Atg12‐Atg5 21, 32. Studies of yeast *Saccharomyces cerevisiae* show that 18 Atg proteins are required for autophagosome formation in higher eukaryotes 32, 33 About half of the Atg members comprise two ubiquitin‐like conjugation systems, the Atg12 and Atg8 systems 32, 34, 35, 36. In the Atg12 system, Atg12 is activated by Atg7 37, 38 and transferred to Atg10 39. But finally, Atg12 irreversibly conjugates to Atg5 and forms the Atg12‐Atg5 complex 37, which strongly enhances the formation of another conjugate, Atg8‐PE, and promotes the autophagy process.

In addition to the inducing effect on the Beclin‐1/Atg7 pathway, our study also suggested the suppressing effect of TAN on Akt/mTOR signaling. Opposing the expression pattern change in Beclin‐1/Atg7 pathway, the levels of total PI3K, p‐Akt, mTOR C1, PDK1, and p‐PDK1 were all inhibited by administration of TAN. As reported previously, blocking of PI3K/Akt/mTOR by stellettin B‐induced autophagy in human non–small cell lung cancer 10. Although the expression pattern of p‐PI3K in this study was distinct from previous results, which was upregulated, it was still indicative of the suppression of downstream effectors of PI3K by TAN administration. This contrary result might be a result of differences in experimental systems and administered agents between the two studies and will be further investigated in the future. The combination of the inducing effect on Beclin‐1/Atg7/Atg12‐Atg5 and the inhibiting effect on PI3K/Akt/mTOR suggests that TAN promotes the autophagy process in OSCC in a multipronged manner.

Interestingly, according to the review of Kang et al. 21, the function of Beclin‐1 in autophagy is also closely related to the PI3K family. Autophagy can also be induced by Beclin 1‐Vps34‐Vps15 core complexes, among which Vps34 forms at least two distinct PI3K complexes in yeast 21. Thus, more exploration was conducted with Beclin‐1 in this study. It was encouraging to find out that knockdown of *Beclin‐1* blocked the effect of TAN on OSCC, both in vitro and in vivo. However, the results also showed that the counteracting effect of *Beclin‐1* knockdown on TAN was only partial. Thus, functioning of TAN might also depend on the activity of other molecules modulating autophagy.

In conclusion, this study clearly showed the antagonizing effect of TAN on OSCC cells. Administration of the agent‐induced caspase‐3‐related apoptosis and autophagy‐dependent cell death in SCC‐9 cells by activating Beclin‐1/Atg7/Atg12‐Atg5 signaling and inhibiting PI3K/Akt/mTOR signaling. Our results also inferred a Beclin‐1‐dependent function of TAN in OSCC cells, although partially. However, our study only provided a preliminary explanation of the mechanism involved in TAN's effect. Data contrary to those in previous reports were also found in our study. Therefore, more comprehensive work is needed in the future to promote the application of the agent.

## Conflict of Interest

No conflict of interest exits in the submission of this manuscript.
